# Prevalence and Risk Factors for Intestinal Protozoan Infections with *Cryptosporidium*, *Giardia*, *Blastocystis* and *Dientamoeba* among Schoolchildren in Tripoli, Lebanon

**DOI:** 10.1371/journal.pntd.0004496

**Published:** 2016-03-14

**Authors:** Marwan Osman, Dima El Safadi, Amandine Cian, Sadia Benamrouz, Céline Nourrisson, Philippe Poirier, Bruno Pereira, Romy Razakandrainibe, Anthony Pinon, Céline Lambert, Ivan Wawrzyniak, Fouad Dabboussi, Frederic Delbac, Loïc Favennec, Monzer Hamze, Eric Viscogliosi, Gabriela Certad

**Affiliations:** 1 Institute Pasteur de Lille, Centre d’Infection et d’Immunitè de Lille (CIIL), UMR CNRS 8204, Inserm U1019, Université de Lille, Biologie et Diversitè des Pathogènes Eucaryotes Emergents (BDPEE), Lille, France; 2 Centre AZM pour la recherche en biotechnologies et ses applications, Universitè Libanaise, Laboratoire de Microbiologie Santè et Environnement (LMSE), Tripoli, Lebanon; 3 Faculté de Santé Publique, Université Libanaise, Beirut, Lebanon; 4 Facultè Libre des Sciences et Technologies de Lille, Universitè Catholique de Lille, Universitè de Lille, Laboratoire Ecologie et Biodiversitè, Lille, France; 5 Clermont Université, Université Blaise Pascal-Université d'Auvergne-CNRS, UMR 6023 Laboratoire Microorganismes: Génome et Environnement, Clermont-Ferrand, France; 6 Laboratoire de Parasitologie-Mycologie, Centre Hospitalier Universitaire Gabriel-Montpied, Clermont-Ferrand, France; 7 CHU Clermont-Ferrand, Unité de Biostatistiques, Direction de la Recherche Clinique, Clermont-Ferrand, France; 8 EA 3800, Université de Rouen & Centre Hospitalier Universitaire Charles Nicolle, Rouen, France; 9 Institute Pasteur de Lille, Unité de Sécurité Microbiologique, Lille, France; 10 Département de la Recherche Médicale, Groupement des Hôpitaux de l’Institut Catholique de Lille, Faculté de Médecine et Maïeutique, Université Catholique de Lille, France; Barcelona Institute for Global Health, SPAIN

## Abstract

**Background:**

Intestinal protozoan infections are confirmed as major causes of diarrhea, particularly in children, and represent a significant, but often neglected, threat to public health. No recent data were available in Lebanon concerning the molecular epidemiology of protozoan infections in children, a vulnerable population at high risk of infection.

**Methodology and Principal Findings:**

In order to improve our understanding of the epidemiology of intestinal pathogenic protozoa, a cross-sectional study was conducted in a general pediatric population including both symptomatic and asymptomatic subjects. After obtaining informed consent from the parents or legal guardians, stool samples were collected in January 2013 from 249 children in 2 schools in Tripoli, Lebanon. Information obtained from a standard questionnaire included demographic characteristics, current symptoms, socioeconomic status, source of drinking water, and personal hygiene habits. After fecal examination by both microscopy and molecular tools, the overall prevalence of parasitic infections was recorded as 85%. *Blastocystis* spp. presented the highest infection rate (63%), followed by *Dientamoeba fragilis* (60.6%), *Giardia duodenalis* (28.5%) and *Cryptosporidium* spp. (10.4%). PCR was also performed to identify species and genotypes of *Cryptosporidium*, subtypes of *Blastocystis*, and assemblages of *Giardia*. Statistical analysis using a logistic regression model showed that contact with family members presenting gastrointestinal disorders was the primary risk factor for transmission of these protozoa.

**Conclusions:**

This is the first study performed in Lebanon reporting the prevalence and the clinical and molecular epidemiological data associated with intestinal protozoan infections among schoolchildren in Tripoli. A high prevalence of protozoan parasites was found, with *Blastocystis* spp. being the most predominant protozoans. Although only 50% of children reported digestive symptoms, asymptomatic infection was observed, and these children may act as unidentified carriers. This survey provides necessary information for designing prevention and control strategies to reduce the burden of these protozoan infections, especially in children.

## Author Summary

Intestinal parasites can infect the gastrointestinal tract of humans. Means of exposure include ingestion of contaminated fruits and vegetables, consumption of infected water and personal contact. Protozoa are considered one of the major groups of parasites. Children are particularly susceptible to infection by these microorganisms, and when they are infected, diarrhea can be the main clinical manifestation. In developing countries, people are at particular risk of infection. However, intestinal parasites, and in particular protozoans, have been taken into account only in a few epidemiological studies. Thus, we conducted an investigation to determine the prevalence, risk factors, and epidemiological information associated with 4 intestinal protozoan infections: *Cryptosporidium*, *Giardia*, *Blastocystis* and *Dientamoeba*, among children attending two schools of Tripoli, Lebanon. A high prevalence of protozoan parasites was found. Although only 50% of children reported digestive symptoms, asymptomatic infection was observed very often, suggesting that these children may act as unknown carriers. In addition, we found that personal contact plays an important role as a risk factor associated with protozoan infection. This epidemiological survey shows the burden of parasitic infections in Lebanese children and provides necessary information to public health authorities for creating prevention and control strategies.

## Introduction

Parasitic infections, and in particular those caused by protozoa, are a major public health problem worldwide. They are among the most widespread human infections in developing countries, with children being the most vulnerable population [1].

In particular, intestinal protozoans, such as *Cryptosporidium* spp. and *Giardia duodenalis* (syn. *G*. *intestinalis* and *G*. *lamblia*), are major causes of diarrhea in children. Transmission of these protozoa is through the oral-fecal route following direct or indirect contact with the infectious stages, including human-to-human, zoonotic, waterborne, and foodborne transmission of both parasites [2], and airborne transmission for *Cryptosporidium* only [2,3]. Additionally, recent data from the Global Enteric Multicenter Study (GEMS) on the burden and etiology of childhood diarrhea in developing countries has shown that the apicomplexan protists *Cryptosporidium* spp. are nowadays one of the leading causes of moderate to severe diarrhea in children aged under 2 years [4,5]. In addition, *Giardia duodenalis* infects approximately 200 million individuals worldwide, and is particularly common among schoolchildren and in daycare centers [6]. In children under 5 years, *G*. *duodenalis* infection may produce severe acute diarrhea. Several studies have also suggested that long-term growth retardation can be a consequence of chronic *Giardia*sis [7].

Because of their significant public health and socioeconomic implications, both parasites *Cryptosporidium* spp. and *G*. *duodenalis* were included in the WHO’s “Neglected disease initiative” in 2004 [8].

Other parasites, such as *Blastocystis* spp. and *Dientamoeba fragilis*, are cosmopolitan protozoans found in the gastrointestinal tract of humans. Nevertheless, the exact contribution of *Blastocystis* spp. and *D*. *fragilis* to pathogenicity has been controversial. The prevalence of *Blastocystis* spp. in humans varies, from 0.5%–24% in industrialized countries to 30%–76% in developing countries [9]. Recently, a *Blastocystis* spp. prevalence of 100% was found in a Senegalese population of children, being the highest prevalence ever reported worldwide for this parasite [10]. All cases were caused by subtypes (STs) 1, 2, 3 and 4, with a predominance of ST3. The prevalence of *D*. *fragilis* ranges from 1% to 52%, according to different geographic regions [11].

Recent studies support the pathogenic nature of both parasites. More than half of the children infected by *Blastocystis* spp. in Senegal presented various gastrointestinal disorders [10], and it is now accepted that the classic clinical features of infection with this parasite include gastrointestinal symptoms such as nausea, anorexia, flatulence, and acute or chronic diarrhea [12]. An association of *Blastocystis* spp. with irritable bowel syndrome (IBS) [13] and extraintestinal manifestations, such as urticaria, has also been suggested [14]. Moreover, invasive and inflammatory potential of the parasite has been reported [15].

Regarding *D*. *fragilis*, infection can be acute or chronic, and symptomatic patients exhibit abdominal pain, persistent diarrhea, loss of appetite, weight loss and flatulence, as well as IBS-like symptoms [16]. Symptoms are observed in 20–58% of infected cases. It has been proposed that *D*. *fragilis* could be a heterogeneous species, with variants having similar morphology but different virulence [17].

In Lebanon, as in other developing countries, intestinal parasitic infections remain responsible for significant morbidity [18,19]. A previous Lebanese study based on microscopic analysis comparing findings for intestinal parasite prevalence at a major tertiary care center between 1997–1998 and 2007–2008 reported the following prevalences: 0% for *Blastocystis* spp., 0.1% for *Cryptosporidium* spp. and 16% for *G*. *duodenalis* in the first period, versus 17% for *Blastocystis* spp., 0% for *Cryptosporidium* spp. and 6% for *G*. *duodenalis* in the second period [20]. Recently, concerning *Blastocystis* spp. and *Cryptosporidium* spp., a prevalence of 19% and 11% respectively, was reported among hospitalized patients after molecular analysis of stool samples [21,22]. Concerning *D*. *fragilis*, no epidemiological data are available to our knowledge. In addition, little information is available in this country on the potential risk factors associated with these protozoan infections in children.

Therefore, the aim of this study was to identify potential risk factors for transmission and to collect molecular epidemiological data on the prevalence and genetic diversity of *Cryptosporidium* spp., *G*. *duodenalis*, *Blastocystis* spp. and *D*. *fragilis* in a population of children attending two schools of different socioeconomic levels in Tripoli, Lebanon.

## Materials and Methods

### Ethics statement

The authorization to conduct this study was obtained from the Lebanese Minister of Public Health (reference number 4–39716). Written informed consents were obtained from the parents or legal guardians of the children, after a clear explanation of the research objectives. This study was conducted in accordance with the Code of Ethics of the World Medical Association (Declaration of Helsinki).

### Questionnaire survey

A standard questionnaire was completed by interviewing the child’s parents or legal guardians, who had given informed consent, in order to obtain a socioeconomic and demographic description including the age, gender, education, residence, occupation and estimated monthly income of the parents, behavioral habits (intake of fruits, vegetables and fast food), health conditions, presence of symptoms (i.e. abdominal pain, diarrhea, vomiting, fever, nausea, headache and discomfort), family members with gastrointestinal disorders, history of previous hospitalizations and medical treatments. Environmental conditions, such as type of water supply, sewage disposal system and presence of domestic animals, were also investigated.

### Study population and collection of samples

**Fig 1:**
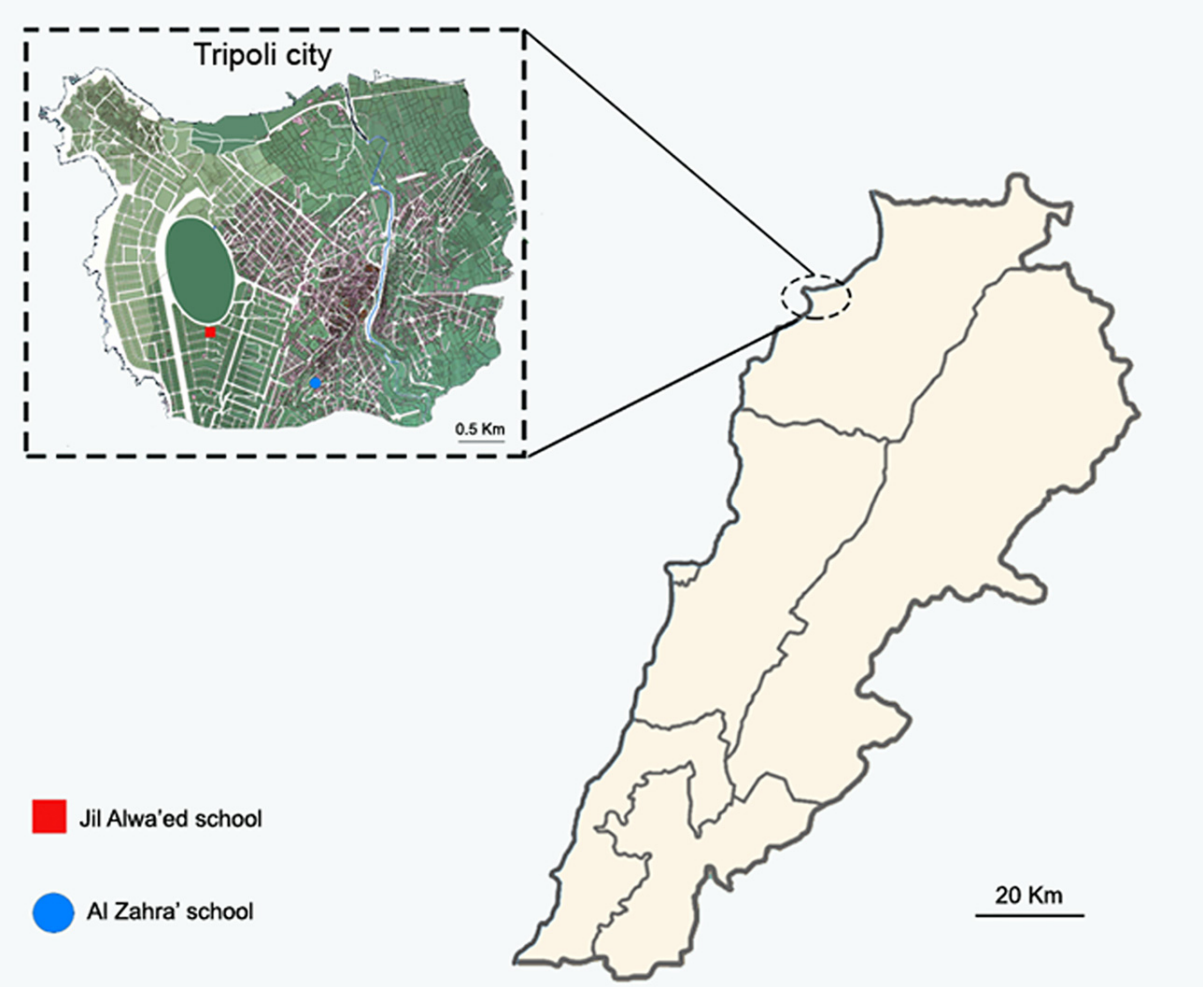
Map of Tripoli, showing the location of Al Zahra’ and Jil Alwa’ed schools.

This cross-sectional study was conducted in Tripoli (latitude 34° 26' 12 N, longitude 35° 50' 58 E), the largest city in northern Lebanon, and the second largest city in the country in terms of demographic and economic importance. The city, situated 85 kilometers (53 miles) north of the capital Beirut, has a Mediterranean climate with mild winters and moderately hot summers. Tripoli’s population is estimated at 500,000 people. Stool samples were collected in 2 nearly schools of different socioeconomic status in Tripoli (Al Zahra’ School and Jil Alwa’ed School) (Fig 1) from two hundred and forty-nine children (149 boys and 100 girls aged between 3 and 16 years) in January 2013. The sample size corresponded to the total number of samples that could be collected for logistical reasons during a specific period of time. The participants were categorized into three groups according to age: under 5 years, between 5 and 9 years and over 9 years, and into two groups according to socioeconomic status: low socioeconomic status (LSES) and high socioeconomic status (HSES). The measure of SES was based on the income, education and occupation of the parents. One fresh stool sample per child was collected in a sterile container and transported immediately to the Department of Microbiology of the AZM Center in Tripoli.

### Parasitological analyses

All stool samples were examined macroscopically, and their characteristics, such as color, consistency, presence of blood, and presence of helminths were recorded. These specimens were also examined by direct-light microscopy (DLM) of wet mounts. For the detection of *Cryptosporidium* spp. oocysts, modified Ziehl-Neelsen (MZN) staining was performed [23], and the slides were examined at 1,000× magnification. For quality control, all examinations were repeated twice by two experienced microscopists. No information was available about potential viral or bacterial infections in these stool samples.

### DNA extraction, species identification and subtyping

All stool specimens were used for molecular detection of *Blastocystis* spp., *Cryptosporidium* spp., *D*. *fragilis* and *G*. *duodenalis*. DNA was extracted from approximately 250 mg of stool samples using the QIAmp DNA Stool Mini Kit (Qiagen GmbH, Hilden, Germany), according to the manufacturer’s recommended procedures. The DNA was eluted in 100 μl of elution buffer (Qiagen) and stored at −20°C until use. The *18S rRNA* detection was performed by nested PCR for *Cryptosporidium* spp. [24] and by real-time PCR for *Blastocystis* spp. [25], *D*. *fragilis* [26] and *G*. *duodenalis* [27], as previously described. To further identify *Giardia* assemblages, the triose-phosphate isomerase (*TPI*) gene was amplified by nested PCR as previously described [28]. *Blastocystis* spp., *Cryptosporidium* spp. and *G*. *duodenalis*-positive PCR products were purified and directly sequenced on both strands by Genoscreen (Lille, France) or Beckman Coulter Genomics (Essex, United Kingdom). The sequences obtained were aligned using the BioEdit v7.0.1 package (http://www.mbio.ncsu.edu/BioEdit/bioedit.html), then compared with gene sequences of these parasites available from the NCBI server (http://www.ncbi.nlm.nih.gov/BLAST/), using the basic local alignment search tool (BLAST). *Blastocystis* spp. STs were identified by determining the exact match or closest similarity against all known STs, according to the updated classification of Alfellani *et al*. [29]. Specimens genotyped as *C*. *parvum* or *C*. *hominis* were further subtyped using nested PCR in order to amplify a fragment of the 60 kDa glycoprotein (*gp60*) gene, as described previously [30].

The amplified DNA fragments were purified and sequenced on both strands, then analyzed by alignment of *gp60* sequences with reference sequences retrieved from GenBank using the ClustalX program (http://www.clustal.org/). *C*. *parvum* and *C*. *hominis gp60* subtypes were named by counting the number of trinucleotide repeats of TCA (A), TCG (G), and TCT (T), and the ACATCA repeat (R) after the trinucleotide repeats [31].

All sequences were uploaded to NCBI GenBank (accession numbers KU311720-KU311975).

### Statistical analyses

Statistical analyses were performed using Stata software, version 13 (StataCorp, College Station, TX, US). The tests were two-sided, with a type I error set at α = 0.05. Quantitative data was presented as the mean ± standard deviation or the median [interquartile range]. The categorical data was presented as frequency and associated proportions. The differences across groups were compared using (1) the Student’s t-test or Mann-Whitney U-test when the conditions of the t-test were not met for continuous variables (assumption of normality studied using the Shapiro-Wilk test and homoscedasticity by the Fisher-Snedecor test), and (2) the chi-squared test or Fisher’s exact test for categorical parameters. Logistic regression models were created to calculate the odds ratios (OR) and 95% confidence interval considering parasite infections as the main outcome. Analyses were based on parasite detection using molecular tools.

## Results

### Prevalence of protozoan infections

A total of 249 schoolchildren (149 male, 100 female) were included in this study. Among them, 157 belonged to the LSES group (mostly children from the Al-Zahra’ School) and the remaining 92 to the HSES group (mostly children from the Jil Alwa’ed School). The age of the participants was between 3 and 16 years (mean age: 10.3 ± 2.7) (Table 1).

**Table 1:** Demographic characteristics of the study population.

	Non-infected children (N = 37)	Infected children (N = 212)
Age (median)	8.48 ± 0.50	9.5 ± 0.21
Gender		
Male	20	129
Female	17	83
Children in the LSES group	19	138
Children in the HSES group	18	74

LSES: low socioeconomic status, HSES: high socioeconomic status

Overall, based on PCR and light microscopy examination, 85% (212/249) of the children were found to be positive for at least one intestinal parasitic infection. Out of a total of 212 infected schoolchildren, the distribution of parasitic infections in males and females was 61% (129/212) and 39% (83/212), respectively. When socioeconomic status was considered, the prevalence was as follows: 65% (138/212) of children in the LSES group and 35% (74/212) in the HSES group. No significant statistical differences regarding parasitic infections related to gender or socioeconomic status were observed. The demographic characteristics of the study population are shown in Table 1.

After molecular analysis of the samples, *Blastocystis* spp. had the highest infection rate (63%), followed by *D*. *fragilis* (60.6%), *G*. *duodenalis* (28.5%) and *Cryptosporidium* spp. (10.4%). As expected, the prevalence of these protozoans was lower in microscopic examination of wet mounts (51.6%, 0%, 14.4%, and 5.6% respectively). Other intestinal parasites were also detected by DLM, as follows: *Entamoeba histolytica/dispar* (5.6%), *Entamoeba coli* (2.4%), *Ascaris lumbricoides* (0.4%), and *Hymenolepis nana* (0.4%).

**Fig 2:**
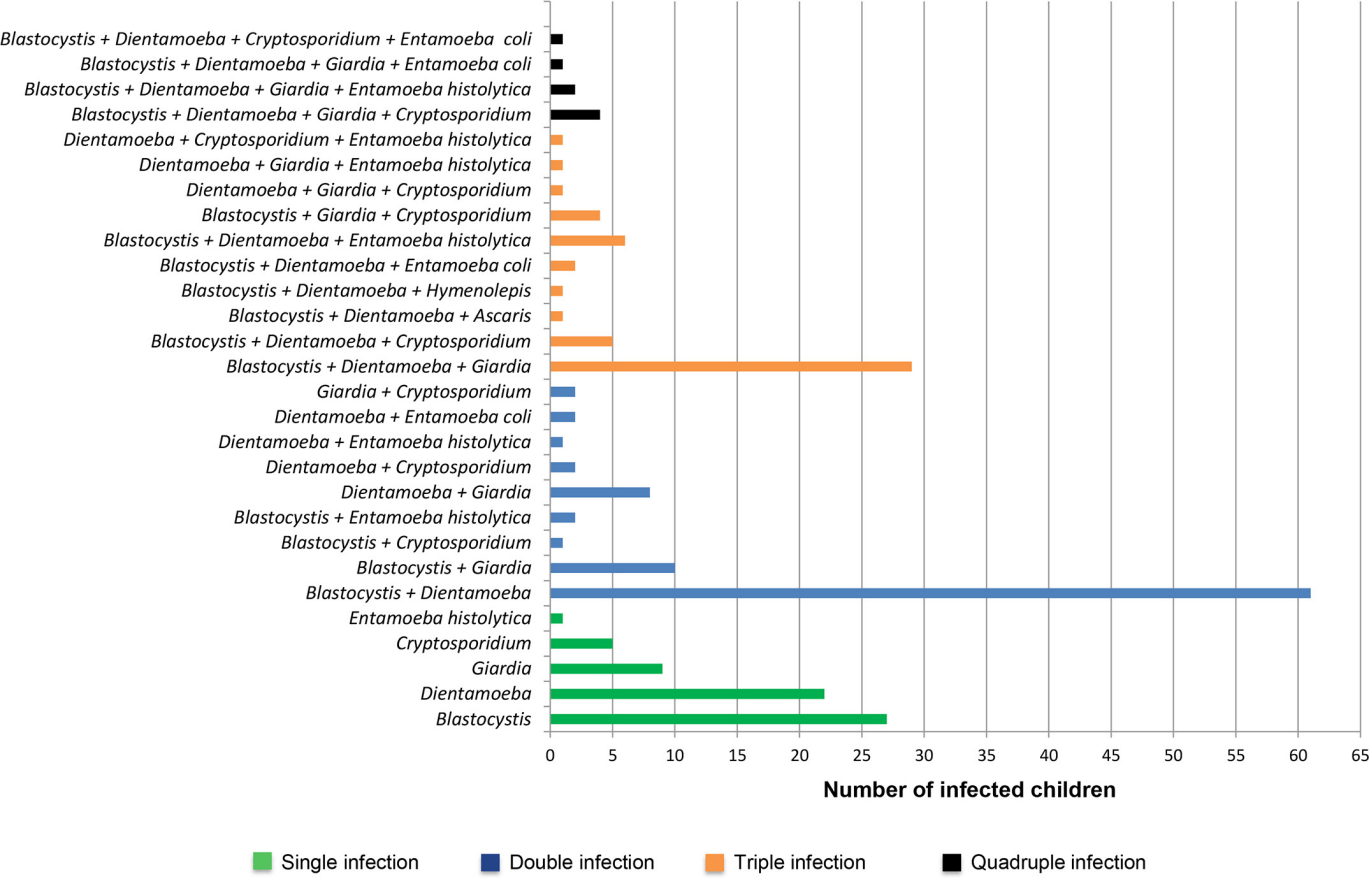
Distribution of single and mixed parasitic infections in schoolchildren in Tripoli. Single, double, triple and quadruple infections are shown. Prevalences of *Blastocystis* spp., *D*. *fragilis*, *Cryptosporidium* spp. and *G*. *duodenalis* are based on molecular diagnosis.

Mixed infections with two parasites were found in 35.7% of children (89/249). The most common dual infection was with *Blastocystis* spp. and *D*. *fragilis*, with a prevalence of 68.5% (61/89). In addition, 11.6% (29/249) of children exhibited triple parasitic infections with *Blastocystis* spp., *D*. *fragilis* and *G*. *duodenalis*. Other cases of mixed infections are shown in Fig 2.

### Clinical manifestations and risk factors for transmission

In total, 125 out of 249 children had symptoms at the time of the survey. Among parasitized children, gastrointestinal symptoms were common (55%). Abdominal pain, diarrhea, vomiting, and fever were reported in 51% (108/212), 28% (60/212), 11% (23/212), and 6% (12/212) of children, respectively. Of the total of 157 *Blastocystis* spp., 151 *D*. *fragilis*, 71 *G*. *duodenalis* and 26 *Cryptosporidium* spp.*-*infected children, 45%, 47%, 69%, and 27% respectively, were asymptomatic.

A logistic regression model was created to identify the risk factors for transmission of these intestinal parasitic infections. The overall presence of abdominal pain (OR: 5.4, CI: 2.1–13.4, P<0.001) and diarrhea (OR: 4.5, CI: 1.3–15.1, P: 0.009), and having members of the same household with gastrointestinal symptoms (OR: 9.6, CI: 2.2–40.9, P<0.001) were significantly predictive of the risk of intestinal parasitic infections in children.

Distribution of protozoan infections among children according to risk factors is shown in Table 2. Univariate logistic regression analysis showed the presence of abdominal pain (OR: 1.9, CI: 1.1–3.2, P: 0.02) and contact with parents having gastrointestinal symptoms (OR: 1.9, CI: 1.0–3.4, P: 0.03) to be the main factors significantly associated with *Blastocystis* spp. infection.

**Table 2:** Distribution of protozoan infections among schoolchildren in Tripoli according to risk factors.

		*Blastocystis* spp.		*Dientamoeba fragilis*		*Giardia duodenalis*		*Cryptosporidium* spp.	
Risk factor		Prevalence *% (N)	*P*-value; OR (IC95%)	Prevalence *% (N)	*P*-value; OR (IC95%)	Prevalence *% (N)	*P*-value; OR (IC95%)	Prevalence *% (N)	*P*-value; OR (IC95%)
**Age**	**< 5 years**	61.5% (8/13)	1.0; 0.93 [0.3–2.9]	53.8% (7/13)	0.77; 0.74 [0.2–2.3]	23.1% (3/13)	0.76; 0.74 [0.2–2.8]	38.4% (5/13)	**0.006; 6.4 [1.9–21.3]**
	**≥ 5 years**	63.1% (149/236)		61% (144/236)		28.8% (68/236)		8.9% (21/236)	
**Sex**	**Male**	66.4% (99/149)	0.18; 1.4 [0.9–2.4]	63.1% (94/149)	0.34; 1.3 [0.8–2.2]	31.5% (47/149)	0.20; 1.5 [0.8–2.6]	10.7% (16/149)	0.85; 1.1 [0.5–2.5]
	**Female**	58% (58/100)		57% (57/100)		24% (24/100)		10% (10/100)	
**Socioeconomic status**	**Low**	66.2% (104/157)	0.18; 1.4 [0.8–2.4]	63.7% (100/157)	0.23; 1.4 [0.8–2.4]	36.3% (57/157)	**<0.001; 3.2 [1.6–6.1]**	10.8% (17/157)	0.83; 1.1 [0.5–2.6]
	**High**	57.6% (53/92)		55.4% (51/92)		15.2% (14/92)		9.8% (9/92)	
**Contact with animals**	**Yes**	36.8% (7/19)	**0.01; 0.3 [0.1–0.8]**	57.9% (11/19)	0.80; 0.9 [0.3–2.3]	15.8% (3/19)	0.20; 0.4 [0.1–1.6]	15.8% (3/19)	0.43; 1.7 [0.5–6.2]
	**No**	65.2% (150/230)		60.9% (140/230)		29.6% (68/230)		10% (23/230)	
**Raw fruit and vegetable consumption**	**Yes**	64.6% (126/195)	0.33; 1.4 [0.7–2.5]	62.1% (121/195)	0.39; 1.3 [0.7–2.4]	32.3% (63/195)	**0.01; 2.7 [1.2–6.2]**	9.7% (19/195)	0.49; 0.7 [0.3–1.8]
	**No**	57.4% (31/54)		55.6% (30/54)		14.8% (8/54)		13% (7/54)	
**Treated water supply in household**	**Yes**	58.3% (35/60)	0.38; 0.8 [0.4–1.4]	56.7% (34/60)	0.47; 0.8 [0.4–1.5]	13.3% (8/60)	**0.003; 0.3 [0.1–0.7]**	13.3% (8/60)	0.40; 1.5 [0.6–3.6]
	**No**	64.6% (122/189)		61.9% (117/189)		33.3% (63/189)		9.5% (18/189)	
**Members of the same household with gastrointestinal symptoms**	**Yes**	72.8% (56/77)	**0.03; 1.9 [1.0–3.4]**	72.8% (56/77)	**0.01; 2.2 [1.2–3.9]**	51.9% (40/77)	**<0.001; 4.9 [2.7–8.9]**	14.3% (11/77)	0.18; 1.7 [0.8–4.0]
	**No**	58.7% (101/172)		55.2% (95/172)		18% (31/172)		8.7% (15/172)	
**Digestive symptoms**	**Yes**	68.8% (86/125)	0.06; 1.6 [0.9–2.7]	64% (80/125)	0.27; 1.3 [0.8–2.2]	42.4% (53/125)	**<0.001;** 4.3 [2.8–8.0]	15.2% (19/125)	**0.01; 3.0 [1.2–7.4]**
	**No**	57.3% (71/124)		57.3% (71/124)		14.5% (18/124)		5.6% (7/124)	
**Abdominal pain**	**Yes**	71.1% (81/114)	**0.02; 1.9 [1.1–3.2]**	65.8% (75/114)	0.13; 1.5 [0.9–2.5]	44.7% (51/114)	**<0.001; 4.7 [2.6–8.5]**	13.2% (15/114)	0.20; 1.7 [0.8–3.9]
	**No**	56.3% (76/135)		56.3% (76/135)		14.8% (20/135)		8.1% (11/135)	
**Diarrhea**	**Yes**	71.4% (45/63)	0.11; 1.7 [0.9–3.1]	60.3% (38/63)	0.95; 1.0 [0.5–1.8]	42.9% (27/63)	**0.004; 2.4 [1.3–4.4]**	22.2% (14/63)	**<0.001; 1.7 [0.8–3.9]**
	**No**	60.2% (112/186)		60.8% (113/186)		23.7% (44/186)		6.5% (12/186)	
**Fever**	**Yes**	46.2% (6/13)	0.24; 0.5 [0.2–1.5]	53.8% (7/13)	0.61; 0.7 [0.2–2.3]	46.2% (6/13)	0.2; 2.3 [0.7–7.0]	38.5% (5/13)	**0.006; 6.4 [1.9–21.3]**
	**No**	64% (151/236)		61% (144/236)		27.5% (65/236)		8.9% (21/236)	
**Vomiting**	**Yes**	63% (17/27)	0.99; 1.0 [0.4–2.3]	59.3% (16/27)	0.88; 0.9 [0.4–2.1]	37% (10/27)	0.37; 1.6 [0.7–3.6]	14.8% (4/27)	0.50; 1.6 [0.5–5.0]
	No	63.1% (140/222)		60.8% (135/222)		27.5% (61/222)		9.9% (22/222)	

*: Diagnosis by molecular biology (nested PCR and real-time PCR)

In the group composed of 151 *D*. *fragilis-*infected children, univariate logistic regression analysis showed that contact with members of the same household having gastrointestinal symptoms (OR: 2.2, CI: 1.2–3.9 P: 0.01) was the only risk factor associated with the presence of this parasite (Table 2). *D*. *fragilis-*infected children were 4 times more likely to be infected with *Blastocystis* spp. (OR: 3.6 CI: 2.1–6.3, P<0.001).

The logistic regression analysis found significant associations between G. *duodenalis* infection and eating raw vegetables and fruits (OR: 2.7, CI: 1.2–6.2, P: 0.01), contact with members of the same household having gastrointestinal symptoms (OR: 4.9, CI: 2.7–8.9, P <0.001), and presence of gastrointestinal symptoms (OR:4.3, CI: 2.8–8.0, P <0.001), such as abdominal pain (OR:4.7, CI:2.6–8.5, P <0.001) and diarrhea (OR:2.4, CI:1.3–4.4, P: 0.004). On the other hand, HSES (OR: 0.3, CI: 0.2–0.6, P<0.001), eating outside of the home (OR = 0.3, CI: 0.1–0.7, P: 0.003), and drinking treated water (OR: 0.3, CI: 0.1–07, P: 0.003) were protective factors against *G*. *duodenalis* infection (Table 2).

The univariate logistic regression analysis showed that children aged under 5 years had a 6 times higher risk of *Cryptosporidium* spp. infection compared with older children (OR: 6.4, CI: 1.9–21.3, P: 0.006). Eating outside of the home (OR: 2.4, CI: 1.1–5.6, P: 0.04) and presence of gastrointestinal symptoms (OR: 3.1, CI: 1.2–7.6, P: 0.01), especially diarrhea (OR: 4.1, CI: 1.8–9.5, P <0.001) or fever (OR: 6.4, CI: 1.9–21.3, P: 0.006), were other factors significantly associated with this infection (Table 2).

### Species identification and subtyping

The real-time PCR products of the 157 samples positive for *Blastocystis* spp. were all sequenced on both strands. With 99% to 100% sequence identity to the reference sequences, 138 isolates corresponded to single infections by one ST, and 3 different STs were identified as follows: ST3 (46.3% of isolates), ST2 (28.3%) and ST1 (25.4%). For the remaining 19 samples, sequence chromatogram analysis revealed the presence of double traces, suggesting mixed infection by different STs that were not identified.

In addition, the PCR products of the 26 samples positive for *Cryptosporidium* spp. were successfully sequenced on both strands. Among them, 20 isolates (77%) were identified as *C*. *hominis*, while 6 isolates (23%) were identified as *C*. *parvum*, all with more than 99% sequence identity to homologous sequences. *Cryptosporidium* spp. other than *C*. *parvum* and *C*. *hominis* were not found. Sequence analysis of the *gp60* gene identified the *C*. *hominis* isolates as belonging to two subtypes: IaA18R3 (4/20) and IbA10G2 (16/20). All of the *C*. *parvum* isolates were identified as the IIaA15G1R1 subtype.

The *Giardia* assemblage was successfully determined by sequencing of the *TPI* gene from 67 of the 71 isolates previously identified by *18 rRNA* PCR. DNA sequencing of the *TPI* gene failed for the 4 others samples. Assemblage B was found in the majority of the samples (64/67), followed by assemblage A (2/67) and a mixed-assemblage infection (1/67).

## Discussion

### Prevalence of protozoan infections

This study demonstrates that protozoan parasitic infections are very common among a community of children living in Tripoli, independent of their socioeconomic status. Such a prevalence is high, considering that the study was performed in an urban area and relied on the collection of a single stool sample per child, instead of the ideal three consecutive samples. A recent study among schoolchildren primarily in rural Malaysia reported a prevalence of parasitic infections of 98% [32].

The most frequent intestinal parasites detected were *Blastocystis* spp. and *D*. *fragilis*, followed by *G*. *duodenalis* and *Cryptosporidium* spp. These 4 protozoans were detected by molecular tools, which are advantageous due to their high sensitivity and specificity. DLM was performed in order to detect co-infection with additional parasites such as helminths, which were identified with a lower prevalence. Although microscopic detection of helminths is widely used as a diagnostic method, microscopy is not very sensitive when infections are light, especially in asymptomatic persons. In addition, specific techniques for the diagnosis of certain nematodes such as *Enterobius vermicularis* were not used.

In the present study, 63% of children were found to be infected with *Blastocystis* spp. after molecular identification. In a previous survey of our group, a lower prevalence of 19% was found in a population of Lebanese symptomatic and asymptomatic patients after microscopic examination of stools [22]. Today, *Blastocystis* spp. is considered an under-reported parasite, with a worldwide distribution and a prevalence far exceeding that of other intestinal parasites in the human population [33,34]. Indeed, its prevalence can reach 100% in developing countries and has been reported at between 1.5% and 20% in industrialized countries [10,33]. The current prevalence of *Blastocystis* spp. among schoolchildren was high, as observed in other countries such as Senegal (100%) [7], Egypt (33%) [35], Syria (28%) [36], the USA (23%) [37], and Pakistan (17%) [38], even if detection methods in these studies are not the same.

Using PCR tools, the prevalence of *D*. *fragilis* reached 61%. A previous study using microscopic techniques reported a prevalence of 38% of *D*. *fragilis* in adult workers in the food sector, in the same geographic area of Lebanon [19]. In addition, in our study, we found a significant association between *Blastocystis* spp. and *D*. *fragilis* co-infection in children (P<0.001). An association between these two protozoans has recently been reported in children presenting gastrointestinal symptoms in the Netherlands [39] and in asymptomatic people in two poor communities in Brazil [40].

*G*. *duodenalis* is one of the most common causes of waterborne disease outbreaks associated with drinking water [41,42]. The prevalence found in our study (29%) is considerably higher than that in other Middle Eastern countries with similar standards of living or in European countries (e.g. Italy, Germany, the UK, Portugal) [43]. In addition, the current prevalence of giardiasis in Lebanon is six times higher than that observed in 2004 (5%) [18]. Nevertheless, the higher sensitivity of molecular tools for the detection of this parasite could likely explain this difference. Even if diagnostic tools were different, recent studies in asymptomatic children around the world reported giardiasis prevalence of 1% in the USA [37], 1% in Italy [44], 1% in the United Kingdom [45], 2% in Germany [46], 7% in Portugal [47], 7% in Pakistan [38], 15% in Syria [36], 16% in Spain [48], 18% in Yemen [49], 32% in Russia [50], and 57% in Cuba [51].

Regarding *Cryptosporidium* spp., this apicomplexan protozoan is one of the most common intestinal parasitic pathogens in the world [52]. Cryptosporidiosis rates are higher in children and immunocompromised patients than in the healthy adult population [53]. However, cryptosporidiosis prevalence varies in different countries: between 1% and 5% in children with diarrhea in developed countries, reaching 49% in developing countries [53,54,55]. Although varying in technical diagnostic tools, the prevalence that we found in children in Lebanon (10%) was in the same range as that observed in Yemen (10%) [31], but lower than that found in others Middle Eastern countries such as Jordan (19%) [56] and Egypt (49%) [55].

Our results based on conventional microscopy showed that infection with *E*. *histolytica/dispar* is prevalent in Lebanon at the present time. Previous studies among presumably older healthy subjects in 2004 reported a prevalence of 2% [57]. It is also more prevalent than in other Middle Eastern countries, such as Syria (0.01%) [36], Qatar (0.3%) [58] and Iran (0.4–2%) [59,60], and in other developed [37] and developing countries [61]. Nevertheless, the parasite is less common than in other developing countries like Pakistan (14%) [38], Yemen (17%) [49], and India (18%) [62]. In a recent study to assess the prevalence and genetic diversity of *E*. *histolytica* in individuals with gastrointestinal symptoms in a rural area of southern Ethiopia, a prevalence of 3.3% was found [63]. The fact that we did not use PCR to detect this parasite strongly suggests that the actual prevalence of these enteric species is likely to be an underestimate.

In a case-control study investigating the prevalence of *Cryptosporidium* spp., *E*. *histolytica* and *G*. *duodenalis* among children < 2 years of age, with and without diarrhea, in Dar es Salaam, Tanzania, an overall high prevalence of these parasites was observed. *Cryptosporidium* spp. infection was more commonly found among young Tanzanian children with diarrhea and *G*. *duodenalis* infection was frequently asymptomatic [64]. Concerning the high prevalence of co-infections of pathogenic and nonpathogenic parasites, our results are comparable to those of other studies [65,66]. The observed polyparasitism could be explained by shared risk factors for parasite infection, such as poor sanitation and hygiene behavior and the fact that the transmission route of these parasites is mainly through the fecal-oral pathway [66].

### Clinical manifestations and associated risk factors

In total, 125 children out of 249 had symptoms at the time of the survey. In relation to the main clinical features of infections, it was found, as expected, that diarrhea was significantly common among *G*. *duodenalis* and *Cryptosporidium* spp.-infected children, but no significant association with this symptom was observed regarding *Blastocystis* spp. or *D*. *fragilis* infections. The interactions and confounding effects that are not evident in a simple comparison of the two groups could also explain the absence of significant associations. Nevertheless, a positive association regarding *Blastocystis* spp. and abdominal pain suggests a pathogenic role for this parasite of controversial clinical significance [67]. Even if children harboring *D*. *fragilis* presented more gastrointestinal symptoms, no significant association was found between this parasite and gastrointestinal disorders in children. Recent studies described that *D*. *fragilis* has struggled to gain recognition as a pathogen, despite the evidence supporting its pathogenic nature [68]. Interestingly, the 124 other children were asymptomatic for protozoan infection and may be carriers responsible for transmission. Consistently, a study among Spanish children attending day care facilities showed that both *G*. *duodenalis* and *Cryptosporidium* spp. infections were asymptomatic in 82% of cases [48].

Concerning the risk factors for protozoan infections, our data analysis found that protozoan parasites could infect both genders in all age groups. However, an age of less than 5 years was significantly associated only with *Cryptosporidium* spp. infection. The reason for this high prevalence is likely due to the immature immunity of young children exposed to this opportunistic parasite [69]. As reported by other authors, no association was found between either gender or age and prevalence of *G*. *duodenalis* infection [47]. It is not yet fully understood why age plays a role in the frequency of *Cryptosporidium* spp. infection, but is not associated with the frequency of giardiasis [70].

Intestinal parasites are usually considered poverty-related diseases [71]. However, no significant association was identified between socioeconomic status and the overall rate of parasitic infections in our study population. Nevertheless, the prevalence of *G*. *duodenalis* was significantly higher in LSES infected children. Interestingly, in a previous study conducted in Peru, *Giardia* spp. and microsporidia were the predominant intestinal parasites among the poorest population, and infections with *Cryptosporidium* spp. were independent of wealth [70]. Furthermore, in our study, only LSES children were infected with helminths (*Ascaris lumbricoides* and *Hymenolepis nana*).

In addition, children who drank untreated water had a 3 times higher risk of infection with *G*. *duodenalis* than those who drank treated water (P: 0.003). Two meta-analyses, including 84 studies in 28 countries, concluded that the quantity of water available to the population in developing countries has more impact on endemic diarrhea cases than water purity itself [72,73]. For the study population in Lebanon, the accessibility of the water supply was not a problem. However, a majority of households did not have a proper sanitary system, favoring fecal contamination via ground seepage, as previously described [74].

The findings of the present study showed that children who had contact with family members presenting gastrointestinal symptoms had a higher risk of infection with these parasites, confirming the direct human-to-human transmission of these protozoans. Thus, the screening and treatment of family members of infected children should be considered for the prevention and control of these infections. Additionally, indirect transmission through contaminated food (raw vegetables and fruits) was found to be a risk factor for giardiasis. In fact, this association is likely due to the fact that fresh vegetables and fruits may be eaten without washing them or with contaminated hands, and it is well known that contaminated hands can play a major role in fecal-oral transmitted diseases [44]. On the other hand, meals outside of the home were significantly associated with *Cryptosporidium* spp. infection.

### Genotyping/subtyping of *Blastocystis* spp., *Cryptosporidium* spp. and *G*. *duodenalis* isolates

The genotyping/subtyping of *Blastocystis* spp., *Cryptosporidium* spp. and *G*. *duodenalis* isolates allows an elucidation of the transmission of these parasites. The majority of *Blastocystis* spp.-positive samples included in this study represented monoinfections (88%) by one ST. Among these positive isolates, three STs were detected as follows: ST3 was the most abundant, followed by ST2 and ST1 (35/138). Our previous study in the Lebanese population also identified the same three STs, with a predominance of ST3 and ST2 [22]. The majority of human *Blastocystis* spp. infections around the world are attributed to ST3 isolates, followed by ST1 and ST2, which is consistent with spread directly from person to person [75]. Interestingly, ST4 was not found in our study. Overall, this ST is common in Europe, but much less frequent in Lebanon as well as in Middle Eastern, African, American and Asian countries [75].

In our cohort of schoolchildren, molecular characterization of *Cryptosporidium* spp. isolates allowed the identification of *C*. *parvum* and *C*. *hominis*, with a predominance of the latter species. It is well known that human cryptosporidiosis is mainly caused by these two species, with *C*. *parvum* considered a zoonotic species while *C*. *hominis* has been mainly associated with anthroponotic transmission [52]. Consistently, a potential secondary transmission of infection among family members was significantly associated with this infection.

These results are consistent with our recent study describing the predominance of *C*. *hominis* in Lebanese hospitalized patients [21]. However, we found different subtypes than those reported in the previous study from our group [21]. Two subtypes belonging to the subtype families Ia and Ib, IaA18R3 and IbA10G2, were identified. The subtype IdA19, which has been described as the predominant subtype in Lebanese hospitalized patients [21], was not found in schoolchildren. The subtype family IbA10G2 has been commonly reported around the world, and is the predominant cause of waterborne outbreaks due to *C*. *hominis* [76]. However, IaA18R3 is a rare subtype recently reported in India and Spain [77,78]. All subtyped *C*. *parvum* isolates were identified as the IIaA15G1R1 subtype. This zoonotic subtype has been reported in both humans and animals in many geographic areas of the world [79]. Moreover, the *C*. *parvum* IIa subtype family has a high genetic diversity, and is responsible for the majority of cryptosporidiosis outbreaks due to *C*. *parvum* [76]. However, the IIc and IId subtype families, which are reported mostly in developing countries, had not been described in Lebanon [55,56,80,81].

Molecular characterization of *G*. *duodenalis* isolates according to *TPI* sequence analysis allowed the identification of assemblages A and B with a large predominance of assemblage B (97%). Both assemblages have been described as zoonotic. However, assemblage B seems to be more human specific [43]. Our results are consistent with other studies among children in other countries such as Brazil, Nepal, and Iran reporting a predominance of assemblage B [82]. Additionally, the association between assemblage occurrence and the age of patients showing higher risk of assemblage B infection in children under 12 years old has been described [83].

### Conclusions

To our knowledge, this is the first study reporting epidemiological data on intestinal protozoan infections among schoolchildren in Lebanon, independent of socioeconomic status. Our results showed a high prevalence of protozoan parasites among this population, *Blastocystis* spp. being the most predominant protozoan. In addition, although 50% of children reported symptoms, many of them were asymptomatic, and these children could serve as unidentified carriers. Contact with family members with gastrointestinal disorders was found to be the main risk factor associated with the presence of protozoan infections. The role of person-to-person contact in the specific transmission of *Blastocystis* spp. and *Cryptosporidium* spp. isolates was consistent with the results of subtyping. The findings of this study provide useful information for the design of prevention strategies, and interventions in target communities at risk.

## Supporting Information

S1 Checklist STROBE checklist. (DOC)

## References

[1] Harhay MO, Horton J, Olliaro PL (2010) Epidemiology and control of human gastrointestinal parasites in children. Expert Rev Anti Infect Ther 8: 219–234. 10.1586/eri.09.119 20109051 PMC2851163

[2] Caccio SM, Thompson RC, McLauchlin J, Smith HV (2005) Unravelling *Cryptosporidium* and *Giardia* epidemiology. Trends Parasitol 21: 430–437. 16046184 10.1016/j.pt.2005.06.013

[3] Sponseller JK, Griffiths JK, Tzipori S (2014) The evolution of respiratory Cryptosporidiosis: evidence for transmission by inhalation. Clin Microbiol Rev 27: 575–586. 10.1128/CMR.00115-13 24982322 PMC4135895

[4] Striepen B (2013) Parasitic infections: Time to tackle cryptosporidiosis. Nature 503: 189–191. 24236315 10.1038/503189a

[5] Kotloff KL, Nataro JP, Blackwelder WC, Nasrin D, Farag TH, et al (2013) Burden and aetiology of diarrhoeal disease in infants and young children in developing countries (the Global Enteric Multicenter Study, GEMS): a prospective, case-control study. Lancet 382: 209–222. 10.1016/S0140-6736(13)60844-2 23680352

[6] Heresi GP, Murphy JR, Cleary TG (2000) Giardiasis. Seminars in Pediatric Infectious Diseases Journal 11: 189–195.

[7] Maikai BV, Umoh JU, Lawal IA, Kudi AC, Ejembi CL, et al (2012) Molecular characterizations of *Cryptosporidium*, *Giardia*, and *Enterocytozoon* in humans in Kaduna State, Nigeria. Exp Parasitol 131: 452–456. 10.1016/j.exppara.2012.05.011 22664352

[8] Savioli L, Smith H, Thompson A (2006) *Giardia* and *Cryptosporidium* join the 'Neglected Diseases Initiative'. Trends Parasitol 22: 203–208. 16545611 10.1016/j.pt.2006.02.015

[9] Wawrzyniak I, Poirier P, Viscogliosi E, Dionigia M, Texier C, et al (2013) *Blastocystis*, an unrecognized parasite: an overview of pathogenesis and diagnosis. Ther Adv Infect Dis 1: 167–178. 10.1177/2049936113504754 25165551 PMC4040727

[10] El Safadi D, Gaayeb L, Meloni D, Cian A, Poirier P, et al (2014) Children of Senegal River Basin show the highest prevalence of *Blastocystis* sp. ever observed worldwide. BMC Infect Dis 14: 164 10.1186/1471-2334-14-164 24666632 PMC3987649

[11] Barratt JL, Harkness J, Marriott D, Ellis JT, Stark D (2011) A review of *Dientamoeba fragilis* carriage in humans: several reasons why this organism should be considered in the diagnosis of gastrointestinal illness. Gut Microbes 2: 3–12. 10.4161/gmic.2.1.14755 21637013

[12] Clark CG, van der Giezen M, Alfellani MA, Stensvold CR (2013) Recent developments in *Blastocystis* research. Adv Parasitol 82: 1–32. 10.1016/B978-0-12-407706-5.00001-0 23548084

[13] Poirier P, Wawrzyniak I, Vivares CP, Delbac F, El Alaoui H (2012) New insights into *Blastocystis* spp.: a potential link with irritable bowel syndrome. PLoS Pathog 8: e1002545 10.1371/journal.ppat.1002545 22438803 PMC3305450

[14] Verma R, Delfanian K (2013) *Blastocystis hominis* associated acute urticaria. Am J Med Sci 346: 80–81. 10.1097/MAJ.0b013e3182801478 23360793

[15] Frealle E, El Safadi D, Cian A, Aubry E, Certad G, et al (2015) Acute *Blastocystis*-associated appendicular peritonitis in a child, Casablanca, Morocco. Emerg Infect Dis 21: 91–94. 10.3201/eid2101.140544 25528951 PMC4285265

[16] Fletcher S, Caprarelli G, Merif J, Andresen D, Hal SV, et al (2014) Epidemiology and geographical distribution of enteric protozoan infections in Sydney, Australia. J Public Health Res 3: 298 10.4081/jphr.2014.298 25343139 PMC4207027

[17] Vandenberg O, Peek R, Souayah H, Dediste A, Buset M, et al (2006) Clinical and microbiological features of dientamoebiasis in patients suspected of suffering from a parasitic gastrointestinal illness: a comparison of *Dientamoeba fragilis* and *Giardia lamblia* infections. Int J Infect Dis 10: 255–261. 16469517 10.1016/j.ijid.2005.05.011

[18] Hamze M, Dabboussi F, Al-Ali K, Ourabi L (2004) [Prevalence of infection by intestinal parasites in north Lebanon: 1997–2001]. East Mediterr Health J 10: 343–348. 16212211

[19] Hamze M, Naja M, Mallat H (2008) [Biological analysis of workers in the food sector in north Lebanon]. East Mediterr Health J 14: 1425–1434. 19161118

[20] Araj GF, Musharrafieh UM, Haydar A, Ghawi A, Itani R, et al (2011) Trends and prevalence of intestinal parasites at a tertiary care center in Lebanon over a decade. J Med Liban 59: 143–148. 22259902

[21] Osman M, El Safadi D, Benamrouz S, Guyot K, Dei-Cas E, et al (2015) Initial data on the molecular epidemiology of cryptosporidiosis in Lebanon. PLoS One 10: e0125129 10.1371/journal.pone.0125129 25950832 PMC4423932

[22] El Safadi D, Meloni D, Poirier P, Osman M, Cian A, et al (2013) Molecular epidemiology of *Blastocystis* in Lebanon and correlation between subtype 1 and gastrointestinal symptoms. Am J Trop Med Hyg 88: 1203–1206. 10.4269/ajtmh.12-0777 23458955 PMC3752823

[23] Henriksen SA, Pohlenz JF (1981) Staining of cryptosporidia by a modified Ziehl-Neelsen technique. Acta Vet Scand 22: 594–596. 6178277 10.1186/BF03548684 PMC8300528

[24] Xiao L, Morgan UM, Limor J, Escalante A, Arrowood M, et al (1999) Genetic diversity within *Cryptosporidium parvum* and related *Cryptosporidium* species. Appl Environ Microbiol 65: 3386–3391. 10427023 10.1128/aem.65.8.3386-3391.1999 PMC91508

[25] Poirier P, Wawrzyniak I, Albert A, El Alaoui H, Delbac F, et al (2011) Development and evaluation of a real-time PCR assay for detection and quantification of *Blastocystis* parasites in human stool samples: prospective study of patients with hematological malignancies. J Clin Microbiol 49: 975–983. 10.1128/JCM.01392-10 21177897 PMC3067686

[26] Stark D, Beebe N, Marriott D, Ellis J, Harkness J (2006) Evaluation of three diagnostic methods, including real-time PCR, for detection of *Dientamoeba fragilis* in stool specimens. J Clin Microbiol 44: 232–235. 16390978 10.1128/JCM.44.1.232-235.2006 PMC1351980

[27] Verweij JJ, Schinkel J, Laeijendecker D, van Rooyen MA, van Lieshout L, et al (2003) Real-time PCR for the detection of *Giardia lamblia*. Mol Cell Probes 17: 223–225. 14580396 10.1016/s0890-8508(03)00057-4

[28] Sulaiman IM, Fayer R, Bern C, Gilman RH, Trout JM, et al (2003) Triosephosphate isomerase gene characterization and potential zoonotic transmission of *Giardia duodenalis*. Emerg Infect Dis 9: 1444–1452. 14718089 10.3201/eid0911.030084 PMC3035538

[29] Alfellani MA, Taner-Mulla D, Jacob AS, Imeede CA, Yoshikawa H, et al (2013) Genetic diversity of *Blastocystis* in livestock and zoo animals. Protist 164: 497–509. 10.1016/j.protis.2013.05.003 23770574

[30] Alves M, Xiao L, Sulaiman I, Lal AA, Matos O, et al (2003) Subgenotype analysis of *Cryptosporidium* isolates from humans, cattle, and zoo ruminants in Portugal. J Clin Microbiol 41: 2744–2747. 12791920 10.1128/JCM.41.6.2744-2747.2003 PMC156540

[31] Alyousefi NA, Mahdy MA, Lim YA, Xiao L, Mahmud R (2013) First molecular characterization of *Cryptosporidium* in Yemen. Parasitology 140: 729–734. 10.1017/S0031182012001953 23369243

[32] Al-Delaimy AK, Al-Mekhlafi HM, Nasr NA, Sady H, Atroosh WM, et al (2014) Epidemiology of intestinal polyparasitism among Orang Asli school children in rural Malaysia. PLoS Negl Trop Dis 8: e3074 10.1371/journal.pntd.0003074 25144662 PMC4140674

[33] Tan KS (2008) New insights on classification, identification, and clinical relevance of *Blastocystis* spp. Clin Microbiol Rev 21: 639–665. 10.1128/CMR.00022-08 18854485 PMC2570156

[34] Alfellani MA, Stensvold CR, Vidal-Lapiedra A, Onuoha ES, Fagbenro-Beyioku AF, et al (2013) Variable geographic distribution of *Blastocystis* subtypes and its potential implications. Acta Trop 126: 11–18. 10.1016/j.actatropica.2012.12.011 23290980

[35] Rayan HZ, Ismail OA, El Gayar EK (2007) Prevalence and clinical features of *Dientamoeba fragilis* infections in patients suspected to have intestinal parasitic infection. J Egypt Soc Parasitol 37: 599–608. 17985591

[36] Al-kafri A, Harba A (2009) Intestinal Parasites in Basic Education Pupils in Urban and Rural Idlb. Syrian Clinical Laboratory Revues 5: 2–5.

[37] Amin OM (2002) Seasonal prevalence of intestinal parasites in the United States during 2000. Am J Trop Med Hyg 66: 799–803. 12224595 10.4269/ajtmh.2002.66.799

[38] Mehraj V, Hatcher J, Akhtar S, Rafique G, Beg MA (2008) Prevalence and factors associated with intestinal parasitic infection among children in an urban slum of Karachi. PLoS One 3: e3680 10.1371/journal.pone.0003680 18997865 PMC2577067

[39] Maas L, Dorigo-Zetsma JW, de Groot CJ, Bouter S, Plotz FB, et al (2014) Detection of intestinal protozoa in paediatric patients with gastrointestinal symptoms by multiplex real-time PCR. Clin Microbiol Infect 20: 545–550. 10.1111/1469-0691.12386 24131443

[40] David EB, Guimaraes S, de Oliveira AP, Goulart de Oliveira-Sequeira TC, Nogueira Bittencourt G, et al (2015) Molecular characterization of intestinal protozoa in two poor communities in the State of São Paulo, Brazil. Parasit Vectors 8: 103 10.1186/s13071-015-0714-8 25889093 PMC4335703

[41] Baldursson S, Karanis P (2011) Waterborne transmission of protozoan parasites: review of worldwide outbreaks—an update 2004–2010. Water Res 45: 6603–6614. 10.1016/j.watres.2011.10.013 22048017

[42] Yoder JS, Wallace RM, Collier SA, Beach MJ, Hlavsa MC (2012) Cryptosporidiosis surveillance—United States, 2009–2010. MMWR Surveill Summ 61: 1–12. 22951493

[43] Ryan U, Caccio SM (2013) Zoonotic potential of *Giardia*. Int J Parasitol 43: 943–956. 10.1016/j.ijpara.2013.06.001 23856595

[44] Guidetti C, Ricci L, Vecchia L (2010) [Prevalence of intestinal parasitosis in Reggio Emilia (Italy) during 2009]. Infez Med 18: 154–161. 20956870

[45] Davies AP, Campbell B, Evans MR, Bone A, Roche A, et al (2009) Asymptomatic carriage of protozoan parasites in children in day care centers in the United Kingdom. Pediatr Infect Dis J 28: 838–840. 10.1097/INF.0b013e31819d646d 19684527

[46] Sagebiel D, Weitzel T, Stark K, Leitmeyer K (2009) Giardiasis in kindergartens: prevalence study in Berlin, Germany, 2006. Parasitol Res 105: 681–687. 10.1007/s00436-009-1438-5 19404678

[47] Julio C, Vilares A, Oleastro M, Ferreira I, Gomes S, et al (2012) Prevalence and risk factors for *Giardia duodenalis* infection among children: a case study in Portugal. Parasit Vectors 5: 22 10.1186/1756-3305-5-22 22284337 PMC3275531

[48] Mateo M, Montoya A, Bailo B, Saugar JM, Aguilera M, et al (2014) Detection and molecular characterization of *Giardia duodenalis* in children attending day care centers in Majadahonda, Madrid, Central Spain. Medicine (Baltimore) 93: e75. 25275524 10.1097/MD.0000000000000075 PMC4616291

[49] Alyousefi NA, Mahdy MA, Mahmud R, Lim YA (2011) Factors associated with high prevalence of intestinal protozoan infections among patients in Sana'a City, Yemen. PLoS One 6: e22044 10.1371/journal.pone.0022044 21789210 PMC3138770

[50] Kramar LV, Reznikov EV, Kramar OG (2003) Prevalence if giardiasis in Volgograd city population. Med Parazitol (Mosk): 38–39. 14727490

[51] Canete R, Diaz MM, Avalos Garcia R, Laud Martinez PM, Manuel Ponce F (2012) Intestinal parasites in children from a day care centre in Matanzas City, Cuba. PLoS One 7: e51394 10.1371/journal.pone.0051394 23236493 PMC3517550

[52] Chalmers RM, Katzer F (2013) Looking for *Cryptosporidium*: the application of advances in detection and diagnosis. Trends Parasitol 29: 237–251. 10.1016/j.pt.2013.03.001 23566713 PMC7106352

[53] ANOFEL (2010) Laboratory-based surveillance for *Cryptosporidium* in France, 2006–2009. Euro Surveill 15: 19642 20739000

[54] Cardona GA, Carabin H, Goni P, Arriola L, Robinson G, et al (2011) Identification and molecular characterization of *Cryptosporidium* and *Giardia* in children and cattle populations from the province of Alava, North of Spain. Sci Total Environ 412–413: 101–108. 10.1016/j.scitotenv.2011.09.076 22030246

[55] Helmy YA, Krucken J, Nockler K, von Samson-Himmelstjerna G, Zessin KH (2013) Molecular epidemiology of *Cryptosporidium* in livestock animals and humans in the Ismailia province of Egypt. Vet Parasitol 193: 15–24. 10.1016/j.vetpar.2012.12.015 23305974

[56] Hijjawi N, Ng J, Yang R, Atoum MF, Ryan U (2010) Identification of rare and novel *Cryptosporidium* GP60 subtypes in human isolates from Jordan. Exp Parasitol 125: 161–164. 10.1016/j.exppara.2010.01.011 20109456

[57] Saab BR, Musharrafieh U, Nassar NT, Khogali M, Araj GF (2004) Intestinal parasites among presumably healthy individuals in Lebanon. Saudi Med J 25: 34–37. 14758375

[58] Abu-Madi MA, Behnke JM, Doiphode SH (2010) Changing trends in intestinal parasitic infections among long-term-residents and settled immigrants in Qatar. Parasit Vectors 3: 98 10.1186/1756-3305-3-98 20946623 PMC2972266

[59] Tappeh Kh H, Mohammadzadeh H, Rahim RN, Barazesh A, Khashaveh S, et al (2010) Prevalence of Intestinal Parasitic Infections among Mentally Disabled Children and Adults of Urmia, Iran. Iran J Parasitol 5: 60–64. 22347245 PMC3279829

[60] Pestehchian N, Nazary M, Haghighi A, Salehi M, Yosefi H (2011) Frequency of *Entamoeba histolytica* and *Entamoeba dispar* prevalence among patients with gastrointestinal complaints in Chelgerd city, southwest of Iran(*). J Res Med Sci 16: 1436–1440. 22973344 PMC3430060

[61] Escobedo AA, Canete R, Nunez FA (2008) Prevalence, risk factors and clinical features associated with intestinal parasitic infections in children from San Juan y Martinez, Pinar del Rio, Cuba. West Indian Med J 57: 377–382. 19566020

[62] Parameshwarappa K, Chandrakanth C, Sunil B (2012) The Prevalence of Intestinal Parasitic Infestations and the Evaluation of Different Concentration Techniques of the Stool Examination. Journal of Clinical and Diagnostic Research 4662:2392.

[63] Flecha MJ, Benavides CM, Tissiano G, Tesfamariam A, Cuadros J, et al (2015) Detection and molecular characterisation of *Giardia duodenalis*, *Cryptosporidium* spp. and *Entamoeba* spp. among patients with gastrointestinal symptoms in Gambo Hospital, Oromia Region, southern Ethiopia. Trop Med Int Health 20: 1213–1222. 10.1111/tmi.12535 25939247

[64] Tellevik MG, Moyo SJ, Blomberg B, Hjollo T, Maselle SY, et al (2015) Prevalence of *Cryptosporidium parvum*/*hominis*, *Entamoeba histolytica* and *Giardia lamblia* among Young Children with and without Diarrhea in Dar es Salaam, Tanzania. PLoS Negl Trop Dis 9: e0004125 10.1371/journal.pntd.0004125 26452235 PMC4599730

[65] Ouattara M, N'Guessan N A, Yapi A, N'Goran E K (2010) Prevalence and spatial distribution of *Entamoeba histolytica*/*dispar* and *Giardia lamblia* among schoolchildren in Agboville area (Côte d'Ivoire). PLoS Negl Trop Dis 4: e574 10.1371/journal.pntd.0000574 20087416 PMC2800181

[66] Hurlimann E, Yapi RB, Houngbedji CA, Schmidlin T, Kouadio BA, et al (2014) The epidemiology of polyparasitism and implications for morbidity in two rural communities of Côte d'Ivoire. Parasit Vectors 7: 81 10.1186/1756-3305-7-81 24568206 PMC3942297

[67] Mehlhorn H, Tan KS, Yoshikawa H (2012) *Blastocystis*: Pathogen or Passenger?: Springer. 225 p.

[68] Stark D, Barratt J, Roberts T, Marriott D, Harkness J, et al (2010) A review of the clinical presentation of dientamoebiasis. Am J Trop Med Hyg 82: 614–619. 10.4269/ajtmh.2010.09-0478 20348509 PMC2844584

[69] Fournet N, Deege MP, Urbanus AT, Nichols G, Rosner BM, et al (2013) Simultaneous increase of *Cryptosporidium* infections in the Netherlands, the United Kingdom and Germany in late summer season, 2012. Euro Surveill 10;18(2). pii: 20348. 23324424

[70] Nundy S, Gilman RH, Xiao L, Cabrera L, Cama R, et al (2011) Wealth and its associations with enteric parasitic infections in a low-income community in Peru: use of principal component analysis. Am J Trop Med Hyg 84: 38–42. 10.4269/ajtmh.2011.10-0442 21212198 PMC3005495

[71] Osei-Atweneboana MY, Lustigman S, Prichard RK, Boatin BA, Basanez MG (2012) A research agenda for helminth diseases of humans: health research and capacity building in disease-endemic countries for helminthiases control. PLoS Negl Trop Dis 6: e1602 10.1371/journal.pntd.0001602 22545167 PMC3335878

[72] Esrey SA, Feachem RG, Hughes JM (1985) Interventions for the control of diarrhoeal diseases among young children: improving water supplies and excreta disposal facilities. Bull World Health Organ 63: 757–772. 3878742 PMC2536385

[73] Esrey SA, Collett J, Miliotis MD, Koornhof HJ, Makhale P (1989) The risk of infection from *Giardia lamblia* due to drinking water supply, use of water, and latrines among preschool children in rural Lesotho. Int J Epidemiol 18: 248–253. 2722373 10.1093/ije/18.1.248

[74] Schmidt M, Al-Nozaily F, Al-Ghorbany A (2008) Standards for and Evaluation of Small-Scale Dam Projects in Yemen. Standards and Thresholds for Impact Assessment. pp. 133–144.

[75] Stensvold CR (2013) *Blastocystis*: Genetic diversity and molecular methods for diagnosis and epidemiology. Trop Parasitol 3: 26–34. 10.4103/2229-5070.113896 23961438 PMC3745667

[76] Chalmers RM (2012) Waterborne outbreaks of cryptosporidiosis. Ann Ist Super Sanita 48: 429–446. 10.4415/ANN_12_04_10 23247139

[77] Sharma P, Sharma A, Sehgal R, Malla N, Khurana S (2013) Genetic diversity of *Cryptosporidium* isolates from patients in North India. Int J Infect Dis 17: e601–605. 10.1016/j.ijid.2012.12.003 23332591

[78] Fuentes I, Martin C, Beristain X, Mazon A, Saugar JM, et al (2014) *Cryptosporidium hominis* genotypes involved in increased incidence and clusters of cases, Navarra, Spain, 2012. Epidemiol Infect: 1–4. 10.1017/S0950268814001836 PMC9507134 25017000

[79] Ryan U, Fayer R, Xiao L (2014) *Cryptosporidium* species in humans and animals: current understanding and research needs. Parasitology 141: 1667–1685. 10.1017/S0031182014001085 25111501

[80] Nazemalhosseini-Mojarad E, Haghighi A, Taghipour N, Keshavarz A, Mohebi SR, et al (2011) Subtype analysis of *Cryptosporidium parvum* and *Cryptosporidium hominis* isolates from humans and cattle in Iran. Vet Parasitol 179: 250–252. 10.1016/j.vetpar.2011.01.051 21376469

[81] Adamu H, Petros B, Zhang G, Kassa H, Amer S, et al (2014) Distribution and clinical manifestations of *Cryptosporidium* species and subtypes in HIV/AIDS patients in Ethiopia. PLoS Negl Trop Dis 8: e2831 10.1371/journal.pntd.0002831 24743521 PMC3990574

[82] El Fatni C, Olmo F, El Fatni H, Romero D, Rosales MJ (2014) First genotyping of *Giardia duodenalis* and prevalence of enteroparasites in children from Tetouan (Morocco). Parasite 21: 48 10.1051/parasite/2014049 25259605 PMC4176428

[83] Mahdy AK, Surin J, Mohd-Adnan A, Wan KL, Lim YA (2009) Molecular characterization of *Giardia duodenalis* isolated from Semai Pahang Orang Asli (Peninsular Malaysia aborigines). Parasitology 136: 1237–1241. 10.1017/S0031182009990527 19660153

