# Correction: Prevalence and Risk Factors for Intestinal Protozoan Infections with *Cryptosporidium*, *Giardia*, *Blastocystis* and Dientamoeba among Schoolchildren in Tripoli, Lebanon

**DOI:** 10.1371/journal.pntd.0004643

**Published:** 2016-04-15

**Authors:** Marwan Osman, Dima El Safadi, Amandine Cian, Sadia Benamrouz, Céline Nourrisson, Philippe Poirier, Bruno Pereira, Romy Razakandrainibe, Anthony Pinon, Céline Lambert, Ivan Wawrzyniak, Fouad Dabboussi, Frederic Delbac, Loïc Favennec, Monzer Hamze, Eric Viscogliosi, Gabriela Certad

There is a spelling error in affiliation 1 for authors Marwan Osman, Dima El Safadi, Amandine Cian, Sadia Benamrouz, Eric Viscogliosi and Gabriela Certad. Affiliation 1 should be: Institut Pasteur de Lille, Centre d’Infection et d’Immunitè de Lille (CIIL), UMR CNRS 8204, Inserm U1019, Université de Lille, Biologie et Diversitè des Pathogènes Eucaryotes Emergents (BDPEE), Lille, France
